# Automated Adherence Reminders for High Risk Children With Asthma: A Research Protocol

**DOI:** 10.2196/resprot.6674

**Published:** 2017-03-27

**Authors:** Sarah A Adams, Michelle Chan Leach, Chris Feudtner, Victoria A Miller, Chén Collin Kenyon

**Affiliations:** ^1^ The Children's Hospital of Philadelphia PolicyLab and Center for Pediatric Clinical Effectiveness Philadelphia, PA United States; ^2^ Sidney Kimmel Medical College Thomas Jefferson University Philadelphia, PA United States; ^3^ Perelman School of Medicine University of Pennsylvania Philadelphia, PA United States; ^4^ The Children's Hospital of Philadelphia Philadelphia, PA United States

**Keywords:** asthma, pediatrics, child, child, preschool, adolescent, metered dose inhalers, medication adherence, text messaging, pilot projects, randomized controlled trial, clinical protocols

## Abstract

**Background:**

The use of inhaled corticosteroid (ICS) medications has been shown to improve asthma control and reduce asthma-related morbidity and mortality. Two recent randomized trials demonstrated dramatic improvements in ICS adherence by monitoring adherence with electronic sensors and providing automated reminders to participants to take their ICS medications. Given their lower levels of adherence and higher levels of asthma-related emergency department (ED) visits, hospitalizations, and death, urban minority populations could potentially benefit greatly from these types of interventions.

**Objective:**

The principal objective of this study will be to evaluate the feasibility, acceptability, and limited efficacy of a text message (short message service, SMS) reminder intervention to enhance ICS adherence in an urban minority population of children with asthma. We will also assess trajectories of ICS adherence in the 2 months following asthma hospitalization.

**Methods:**

Participants will include 40 children aged 2-13 years, who are currently admitted to the Children’s Hospital of Philadelphia (CHOP) for asthma, and their parent or legal guardian. Participants will be assigned to intervention and control arms using a 1:1 randomization scheme. The intervention arm will receive daily text message reminders for a 30-day intervention phase following hospitalization. This will be followed by a 30-day follow-up phase, in which all participants may choose whether or not to receive the text messages. Feasibility will be assessed by measuring (1) retention of the participants through the study phases and (2) perceived usefulness, acceptability, and preferences regarding the intervention components. Limited efficacy outcomes will include percent adherence to prescribed ICS regimen measured using Propeller Health sensors and change in parent-reported asthma control. We will perform an exploratory analysis to assess for discrete trajectories of adherence using group-based trajectory modeling (GBTM).

**Results:**

Study enrollment began in December 2015 and the intervention and follow-up phases are ongoing. Results of the data analysis are expected to be available by December 2016.

**Conclusions:**

This study will add to the literature by providing foundational feasibility data on which elements of a mobile health text-message reminder intervention may need to be modified to suit the needs and constraints of high-risk urban minority populations.

**Trial Registration:**

Clinicaltrials.gov NCT02615743; https://www.clinicaltrials.gov/ct2/show/study/NCT02615743 (Archived with WebCite at http://www.webcitation.org/6ji59rAXN)

## Introduction

Asthma is the most common chronic medical condition in children in the United States, with 8.3% of children affected [1]. Racial disparities have long existed in both asthma prevalence and morbidity. Several reports from the last few years have demonstrated progress in narrowing the disparities gap in both asthma prevalence [1] and morbidity [2]. Despite this progress, black children with asthma still suffer significantly higher rates of asthma emergency department (ED) visits, hospitalizations, and death compared with their white peers [2].

Use of inhaled corticosteroid (ICS) medications has been shown to improve asthma control and reduce asthma-related exacerbations and hospitalizations [3-5]. Despite this evidence, average adherence to prescribed regimens for ICSs in childhood asthma is only around 50% of prescribed doses [6]. Several studies have demonstrated that adherence rates in urban minority populations in the United States are substantially lower, ranging from 11-37% [7-10]. Yet average rates may be misleading, as research from other studies indicates that patterns of day to day medication adherence are highly variable and these patterns change over time [11-13]. These studies capture the inter- and intra-individual variation in medication use and provide valuable information on the kinetics of different adherence trajectories [11-13]. Such studies may help identify the reasons behind adherence lapses, which, in turn, would allow providers to enhance and better tailor adherence intervention strategies.

Prior interventions to enhance daily medication adherence in children with chronic disease have generated modest, at best, improvements in adherence to recommended regimens [14-17]. However, 2 recent randomized trials from Australia and New Zealand demonstrated dramatic improvements in ICS adherence by monitoring adherence with electronic sensors and providing automated messages to remind participants to take their ICS medications [18,19]. Given their lower levels of adherence, urban minority populations could potentially benefit greatly from these types of interventions. Whether reminder-based interventions are feasible and effective in vulnerable urban populations in the United States, however, is unknown. The factors that drive nonadherence in inner city children with asthma appear more complicated than “just forgetting” [6,20,21]. For this reason, dedicated study of the feasibility and efficacy of automated reminder-based interventions may provide valuable information on how to design and tailor future interventions for this population.

The principal objective of this study will be to evaluate the feasibility, acceptability, and limited efficacy of daily text message (short message service, SMS) reminders as a means to improve adherence to ICS prescriptions in urban minority children with asthma in Philadelphia. We will also assess the longitudinal trajectories of ICS use following hospitalization and explore associations with factors previously associated with nonadherence.

## Methods

### Overview of Study

This is a pilot randomized controlled trial to assess the feasibility, acceptability, and limited efficacy of text message reminders for daily ICS medication use in high risk children with asthma. Subjects will include children seen in the ED or hospitalized for asthma at the Children’s Hospital of Philadelphia (CHOP) and their families. Propeller Health electronic sensors will be placed on ICS metered dose inhalers (MDI) at the time of ED or hospital discharge to measure subsequent medication use in both the intervention group and the regular care control group. The intervention group will receive text message reminders delivered to the caregiver’s mobile phone for 30 days following hospitalization. The protocol for the conduct of this study was approved by the CHOP institutional review board and is registered on ClinicalTrials.gov (NCT02615743).

### Setting

Subjects will be recruited from the ED and general pediatrics inpatient units at CHOP. CHOP is a large, free-standing, tertiary care children’s hospital that also serves as a community hospital for the children of Philadelphia, particularly in the west and southwest regions of the city. There are nearly 6000 ED visits and 2500 inpatient admissions for asthma at CHOP each year.

### Participants – Inclusion and Exclusion Criteria

Participants will include 40 children aged 2 to 13 years and their parent or legal guardian. To be enrolled in the study, children and their parent or legal guardian must meet the following eligibility criteria: (1) they must be receiving care for asthma on the ED or inpatient asthma pathways; (2) they must have been prescribed a daily ICS or combined ICS or long-acting beta agonist (LABA) in the year prior to the current admission and the ICS prescribed on discharge must be an MDI compatible with the electronic monitoring sensor (compatible ICS and ICS or LABA medications include Flovent [fluticasone], QVAR [budesonide], Advair MDI [fluticasone-salmeterol], Dulera [mometasone-formoterol], and Asmanex [mometasone-furoate]); (3) children and families must live or receive primary care in Philadelphia ZIP codes with the highest child asthma morbidity (greater than 100 pediatric asthma hospitalizations per year, according to Pennsylvania Health Care Cost Containment Council [22] hospitalization data); and (4) the parent or legal guardian must have a mobile phone plan with unlimited text messages.

Children will be excluded from the study if they are prescribed a controller inhaler to which the electronic device cannot affix (eg, any dry powder inhaler, Symbicort [budesonide or formoterol], or Seretide [fluticasone-salmeterol]) as their primary controller medication. Children with comorbid conditions that may influence the treatment of asthma (such as chronic lung disease, structural heart disease, or cystic fibrosis) will be excluded. Families will be excluded from the study if they have active state social services involvement or if they are non-English speaking. Subjects with significant developmental delays will also be ineligible.

### Outcome Measures

Given that this is a pilot study to assess the feasibility of a text message–based electronic adherence intervention in a vulnerable population, the primary outcomes will be feasibility oriented [23]. Feasibility outcomes will include (1) passive retention through the study and (2) perceived usefulness, acceptability, and preferences regarding the intervention components. Limited efficacy outcomes will include change in parent-reported asthma control and difference in average ICS adherence between the intervention and control conditions. Thirty-day readmissions and parent-reported adherence will be included as secondary outcome measures. Finally, medication use trajectories following hospitalization will be assessed using group-based trajectory modeling.

#### Feasibility

Feasibility will be assessed in 2 ways: (1) passive retention of participants in the intervention and follow-up phases, indicated by ongoing electronic sensor syncing and (2) text message and its usefulness, acceptability, and preferences, assessed via questionnaire. To assess passive retention, we will monitor duration of the interval that the electronic sensor synced with its receiver (either a mobile phone app or cellular hub). In order to sync effectively, the Bluetooth-enabled sensor must be within range of a functioning (plugged in) cellular hub or an open mobile phone app with Bluetooth enabled. Thus, passive retention represents whether the receiver was capable of transmitting MDI actuations from the sensor during the intervention and follow-up phases of the study. Reasons for missing actuation data will be collected during the follow up calls and during individual calls for participants whose sensors fail to sync within the first 2 days of the intervention phase of the study.

**Figure 1:**
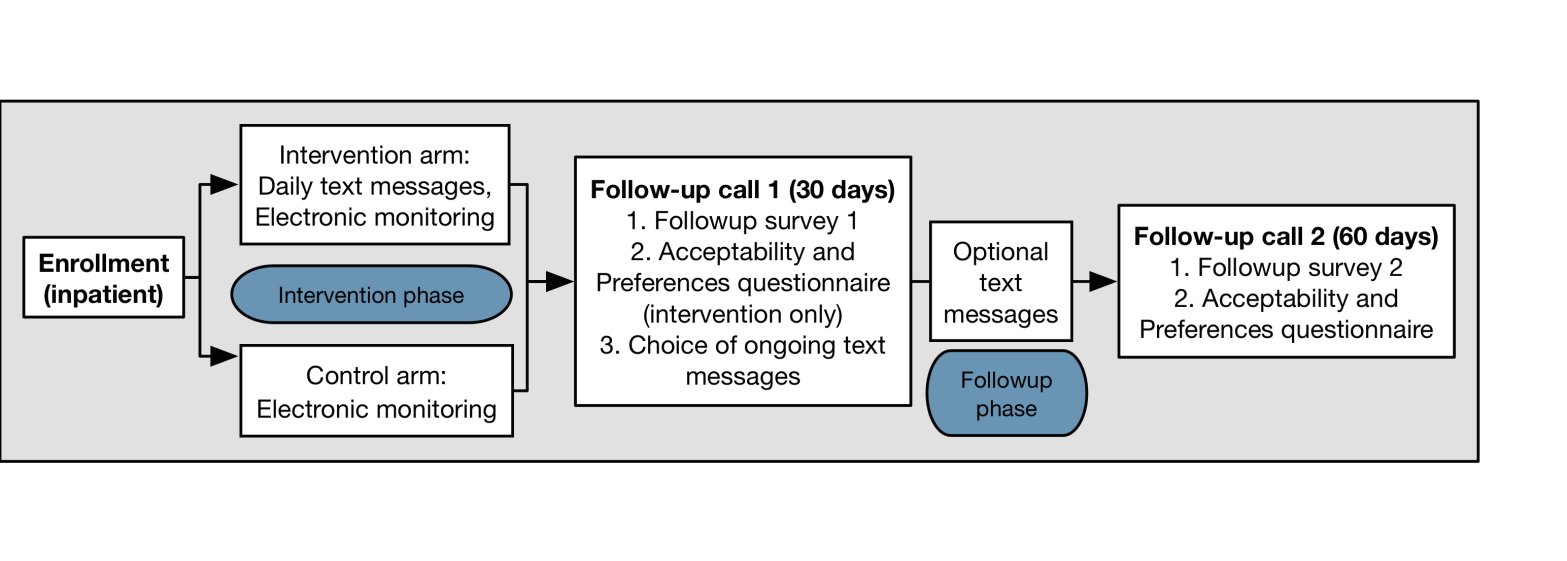
Study flow diagram.

Perceived usefulness, acceptability, and intervention preferences will be measured using a 14-item questionnaire developed de novo for this study and delivered to those who receive the intervention either at follow-up call 1 or 2 (See Figure 1). This survey focused primarily on usefulness and acceptability of the daily text messages, as our prior work in a similar population found electronic sensors to be generally acceptable [24] (see Multimedia Appendix 1).

#### Limited Efficacy

Limited efficacy outcomes for this study will include (1) percent ICS adherence, as measured by electronic monitoring and (2) average change in parent-reported asthma control, assessed using the parent-reported portion of the child Asthma Control Test [25]. Inhaled controller medication use will be measured in both groups using electronic sensors (see description below) affixed atop of the MDI canister. ICS adherence will be calculated from the daily medication use data as the average percent adherence (observed actuations or prescribed actuations) for the intervention group versus the control group for the 30-day intervention phase. The average change in the parent-reported portion of the child Asthma Control Test score between enrollment and the 30-day follow-up call will be calculated for the intervention and control groups.

#### Adherence Trajectories

Finally, we will perform an exploratory analysis to assess for discrete trajectories of adherence using group-based trajectory modeling (GBTM). This analysis will include data from both the intervention and follow-up phases of the study. If no significant differences exist in limited efficacy outcomes between the intervention and control groups, data from both groups will be combined to produce pooled trajectory outcomes.

### Covariates

**Figure 2:**
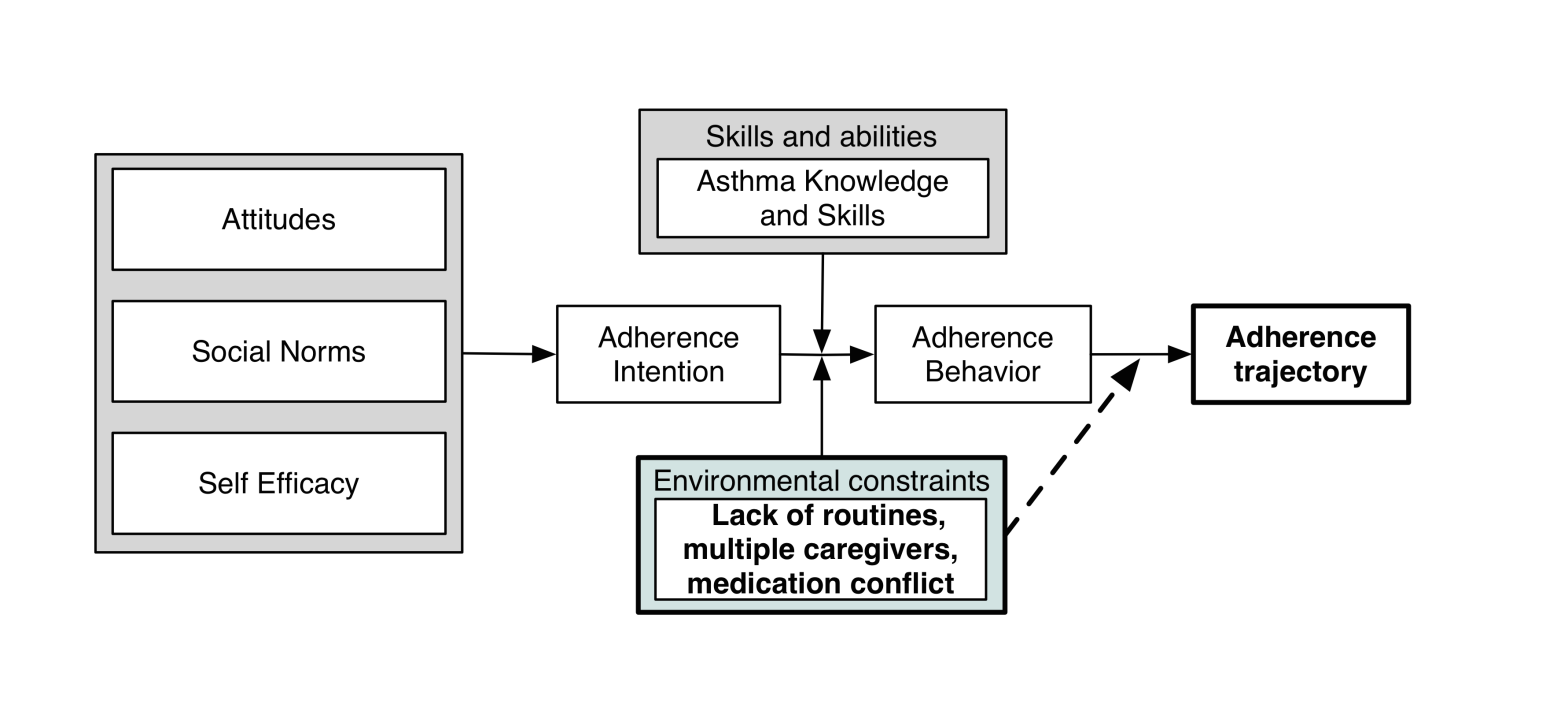
Conceptual framework adapted from the Integrated Behavioral Model.

Factors previously shown to be associated with adherence will be assessed using surveys administered to the caregiver upon enrollment and at 30 and 60 days. We adapted a version of the Integrated Behavior Model [26] as a unifying conceptual framework to contextualize these factors that may be influencing adherence patterns. These include medication beliefs, social norms, self-efficacy, intention to adhere to controller medications, asthma knowledge and skills, environmental constraints (routines, responsibility, and conflict over medication taking), and demographics (see Figure 2).

The Beliefs About Medicines Questionnaire [27] will be used to assess medication beliefs (perceived necessity and concerns regarding ICS use). Descriptive and injunctive social norms will be assessed with separate questions. We will use question blocks from the Caretakers Expectation Scale [28] to assess perceived importance of asthma-related tasks and self-efficacy for each task. The caregiver will be asked to estimate both prior adherence in the previous four weeks, as well as adherence intention for the next 4 weeks. The Asthma Knowledge Questionnaire [29] will assess a basic caregiver understanding of asthma symptoms, triggers, and treatments. The Asthma Routines Questionnaire [30] will measure the strength of routines surrounding asthma care. The Asthma Responsibility Questionnaire [31,32] will assess the degree to which children and caregivers share responsibility for managing asthma. Parent-child conflict over medication use will be measured with 2 questions developed by the study team (see Multimedia Appendix 2).

Demographics will include the caregiver’s relationship to the child, age, marital status, family income level, education level, and whether or not he or she is the child’s primary caregiver. Child demographic information will include the child’s race, ethnicity, age, and number of nights per week he or she typically stays with the primary caregiver.

### Intervention

#### Enrollment

Potential subjects will be screened through the daily census listings for the ED and general pediatrics inpatient units at CHOP, using the protocol inclusion and exclusion criteria. Parents of eligible participants will be approached for informed consent, screening, and enrollment prior to the patient’s discharge from the hospital. Child assent will be obtained from children ages 7 years and older, unless the child is judged to be incapable of assent. If subjects provide consent and enroll, they will complete the initial study visit prior to hospital or ED discharge. As part of enrollment, caregivers will complete an initial 30-minute survey on the Way to Health (WTH) platform (see below for description). Prior to survey completion, participants will be randomized into either the control or intervention group using a 1:1 randomization scheme, stratified by location (ED or inpatient). Each participant will be compensated US $20 through a reusable debit card following completion of each survey, for a total of US $60 for those completing the entire study.

Following the baseline survey, a study team member will attach an electronic sensor to the participant’s ICS inhaler. If the participants do not have a currently filled ICS prescription with them in the hospital room, they will be instructed on how to attach the sensor to the child’s inhaler when they get home. Participants whose sensors are not attached to the inhaler at discharge will be contacted by phone to achieve an initial sync. Participants who have mobile phones will download the Propeller mobile phone app for data transmission purposes, but the participants will not have access to any information from the app. Preferences for the text messages, which are delivered to the parents’ mobile phone via the Way to Health platform (time of day and preferred phone number), will be collected for all participants. Participants will be instructed to use the inhaler as directed by their physician and that they may begin to receive automated text message reminders regarding their child’s medication use.

#### Intervention Phase

The randomized intervention begins the day of discharge from the hospital and lasts 30 days. During this time, the intervention group will receive daily automated text message reminders from the WTH platform at a time of their choosing. In addition to daily text message reminders for the intervention group, both the intervention and control groups will receive programmed text messages when they reach milestones in the study, such as survey or intervention completion. These text messages will also serve as prompts to contact study staff and complete their next survey. The research team will also program text messages reminding participants to continue syncing their electronic monitoring sensor with its receiver by turning on their Bluetooth, if they are using a mobile phone, or by making sure that their hub is plugged in, if not. A total of 3 syncing reminders will be sent over the course of the intervention. Study staff will send individualized text messages or call participants if the sensor fails to sync in the first 2 days of the intervention phase. Researchers will walk the participant through the syncing process and record any reasons for missing data.

At the end of the 30-day period, families will receive a text message instructing them to set up a follow-up call within the next few days. If the participants don’t call within 2-4 days, they will be contacted by the research team. The first follow-up call includes a 10-minute survey for all participants and the questionnaire assessing acceptability and preferences for participants in the intervention arm. After completing the surveys, participants will be asked to choose whether or not to receive text message reminders during the follow-up phase of the study. Automated text messages will be turned on or off for each individual through WTH. All participants will be asked whether they have run out of ICS medication and if they had any problems using the electronic sensor. Any problems that may lead to missing data will be recorded. The participant will then be reminded to transfer the electronic sensor to their new inhaler if or when the prescription is refilled.

#### Follow-up Phase

The follow-up phase begins the day the first follow-up call is completed and continues until 60 days following the start of the randomized intervention. The follow-up phase lasts up to 30 days. Participants will be given 2 weeks following the end of the follow-up period to complete the second follow-up call.

At 60 days, medication adherence monitoring and optional text message reminders will cease and families will receive a message from the study team to set up a second follow-up call (the study completion call) within the next few days. If the participants don’t call within 2-4 days, the research team will contact them. The second follow-up call includes a 10-minute survey for all participants. Participants will be asked again whether they have run out of ICS medication or if they had any problems using the electronic sensor and responses will be recorded. Participants initially assigned to the control group who elected to receive text messages during the follow-up phase will also complete the acceptability and preferences questionnaire.

### Way to Health Platform

**Figure 3:**
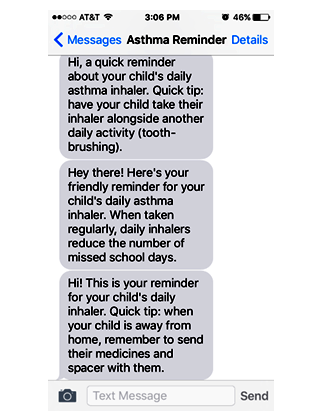
Examples of intervention text messages.

Way to Health (WTH) is a Web-based behavioral intervention toolbox, which automates many of the research functions necessary for conducting randomized controlled trials (waytohealth.org). For this study, the WTH platform will be customized to enroll participants, automate randomization and text message delivery, and complete and store survey data. For participants in the intervention arm, 7 rotating text messages will be programmed into WTH. Each text message will include a reminder to administer the ICS inhaler as well as an asthma-related tip or fact (Figure 3). Each family will be able to customize the time the text message reminders are sent based on caregiver preference.

The Way to Health Platform protects patient information by using a secured firewall accessible only to study research, analysis, and IT staff by use of a password.

### Propeller Health System

We will use Propeller Health electronic monitoring sensors to monitor ICS medication use (propellerhealth.com). Propeller Health sensors record the date, time, and number of inhaler actuations. The Propeller Health sensors have been validated and used in previous studies [33,34].

Families with a mobile phone will use the sensor in conjunction with a research “control” version of Propeller Health’s mobile app, which receives the medication use data from the sensor through the mobile phone’s Bluetooth functionality. Unlike the full Propeller Health mobile app, which provides medication usage summaries, adherence, control status, air quality information, education and personalized trigger information to the participant, the research version provides only medication use and timing data to an encrypted server accessible to the research team alone. Families without a mobile phone will be provided an electronic monitoring device with a paired cellular hub. The hub transmits medication use data from the device to the encrypted server through cellular networks and must be plugged into an electrical outlet for transmission.

The communication between the electronic monitoring sensors and the Propeller Health server is referred to as syncing. Syncing transmits data on inhaler actuations, which refers to medication usage recorded by the sensor. One actuation translates to one single use or puff of the participant’s daily controller medication. The Propeller Health research app allows study staff to see when each participant’s electronic monitoring sensor last synced with the server. However, it does not display data on device actuations. Thus, study staff will know if a participant’s sensor is syncing, but will be blind to each participant’s medication usage data until the end of the intervention interval. The Propeller Health platform will not be used to store protected health information.

### Data Analysis

#### Feasibility

Response rate will be defined as number of participants who complete enrollment, out of the total number of eligible patients who we approach. Passive retention will be evaluated using the median number of days for which the sensor syncs with its mobile phone or hub. Qualitative reasons for missing actuation data (eg, logging out of the app, failing to plug in the hub, or prescription changes) will be recorded if described by the caregiver during follow up or syncing issue calls described above. We will use summary statistics (mean, interquartile range) to describe Likert-based usability, acceptability, and preferences question responses. Qualitative responses regarding what participants liked most about the study and what would make it better will be evaluated for the most common responses.

#### Limited Efficacy

Bivariate testing will be conducted to assess whether the intervention and control groups are balanced on key characteristics, including demographics, baseline asthma control (based on Child Asthma Control Test score on enrollment), and baseline medication adherence. If the groups are balanced, differences in 30-day adherence proportions and parental perceptions of asthma control between the intervention and control groups will be assessed using *t* tests or Wilcoxon Mann-Whitney tests depending on the distribution of the data. If the groups are unbalanced, regression models will be used to adjust for the unbalanced measured confounders.

#### Trajectories

We will use GBTM to explore daily longitudinal adherence data for different adherence patterns over time. We will specify several models with the best fit using 3, 4, 5, and 6 group solutions, based on quadratic trajectories and a normal probability distribution. We will compare the models to identify the model and number of groups that best fit the data based on the lowest Bayesian information criterion (BIC) and group percentages that are sufficiently large (eg, >5% of the population). These trajectory groups will be used to assess qualitative trends in medication use that may merit further study.

## Results

Study enrollment began in December 2015 and the intervention and follow-up phases are ongoing. Results of the data analysis are expected to be available by December 2016.

## Discussion

### Contribution to the Literature

This pilot study will evaluate the feasibility, acceptability, and limited efficacy of a text message reminder intervention to improve ICS adherence in an urban minority population of children with asthma. The study will also assess trajectories of ICS adherence in the 2 months following asthma hospitalization to explore how, when, and why ICS use decays following an acute exacerbation and how this may vary between different families.

While there is a substantial body of literature investigating the reasons for poor adherence to asthma controller medications [3-5], traditional approaches to improving adherence have demonstrated inconsistent results [14-17]. New reminder systems that leverage mobile technology have demonstrated promise in improving adherence in certain populations [18,19,35], but how these technologies and interventions may work in cohorts with the highest levels of asthma morbidity and death has not been thoroughly investigated. This study will add to the literature by providing foundational feasibility data on which components of a mobile health text-message reminder intervention are acceptable and useful and which components may need to be modified to suit the needs and constraints of high risk populations.

### Limitations

We anticipate several limitations of this study. Since the study is oriented to feasibility, the study is not powered to detect between-groups differences in adherence or adherence trajectories. Differences in actuation data, parent perception of asthma control, and medication use trajectories will be evaluated to assess trends for future efficacy testing.

Given the vulnerable nature of the patient population, researcher’s ability to contact caregivers for follow-up calls will limit both completion of the intervention and follow-up surveys and ongoing syncing. Many parents have changing work schedules that make it difficult to predict availability for follow-up calls in advance. Parents also frequently change their primary phone numbers, which limits follow-up and may affect whether they receive the text message intervention for the entire study period. We will reduce this limitation by asking participants for multiple contact numbers and recording preferred times to receive calls.

Most families in this study will go home with a new ICS prescription at discharge, however, the prescription is not always filled at the on-site pharmacy, so the sensor will not always be applied by study staff. In this circumstance, the caregiver must successfully fill the prescription at their pharmacy and correctly attach the electronic sensor themselves in order for actuation data to be available. To address this concern, research staff will conduct reminder calls in the few days following discharge. As a result of this limitation, actuation data at the front end of the intervention phase may be missing for some participants.

## Appendix

Multimedia Appendix 1 Acceptability and preferences questionnaire.

Multimedia Appendix 2 Parent child conflict survey items.

Multimedia Appendix 3 EHealth Consort checklist version 1.6.1.

## Abbreviations

BIC: Bayesian information criterion
ED: emergency department
GBTM: group-based trajectory modeling
ICS: inhaled corticosteroid
LABA: long-acting beta agonist
MDI: metered dose inhalers
SMS: short message service
WTH: Way to Health
