# Supplementary material for: Automated Adherence Reminders for High Risk Children With Asthma: A Research Protocol
Source: JMIR Res Protoc. 2017 Mar 27;6(3):e48. doi: 10.2196/resprot.6674 (PMC5387114; doi:10.2196/resprot.6674)
Supplement: Multimedia Appendix 1 [file resprot_v6i3e48_app1.pdf]

Acceptance and Preferences

Text Message Preferences Survey

The following questions will ask about how you felt about the text messages you received during this study.

There are no right or wrong answers. If you have any questions, please ask the research assistant for help.

Hey Mikki: Was this person assigned to receive text messages in the last 30 days?

A
yes

B
no

I received a text message reminder every day.

A
Yes

B
No

The text messages were easy to understand.

Strongly Agree	Agree	Neutral	Disagree	Strongly Disagree

The text messages came too often.

Strongly Agree	Agree	Neutral	Disagree	Strongly Disagree

The text messages were helpful to avoid missed doses.

Strongly Agree	Agree	Neutral	Disagree	Strongly Disagree
○	○	○	○	○

I found the text messages to be annoying.

Strongly Agree	Agree	Neutral	Disagree	Strongly Disagree
○	○	○	○	○

I didn't usually read the text messages.

Strongly Agree	Agree	Neutral	Disagree	Strongly Disagree
○	○	○	○	○

I would recommend this program to other families with children with asthma.

Strongly Agree	Agree	Neutral	Disagree	Strongly Disagree
○	○	○	○	○

If given the choice, I would like to continue to receive texts like these in the future.

Strongly Agree	Agree	Neutral	Disagree	Strongly Disagree
○	○	○	○	○

I would prefer to create my own reminder text message.

Strongly Agree	Agree	Neutral	Disagree	Strongly Disagree
○	○	○	○	○

I would prefer to see a report of my child's medication use (on a website or an email).

Strongly Agree	Agree	Neutral	Disagree	Strongly Disagree
○	○	○	○	○

I think that having an incentive (a game or reward) for taking medication would improve my child's medication use.

Strongly Agree	Agree	Neutral	Disagree	Strongly Disagree
○	○	○	○	○

What is the best way to give you information about your child's asthma and asthma care, beyond what you discuss in your appointments with your medical providers?

A
Text Messages

B
Smartphone App

C
Website

D
Written Materials

E
Phone Conversation

F
In-Person Conversation

What did you usually do when you received a text message when your child was with you?

A
Asked my child if he or she had taken the controller medication

B
Gave my child the controller medication

C
Nothing - I didn't read it

D
Nothing - I read it but it often came after or too far before the medication was due

E
Nothing - I read it but didn't think my child needed the medicine

What did you usually do when you received a text message when your child was **not** with you?

A
Nothing - I didn't read it

B
Read it but did not forward it to an adult with the child

C
Forwarded the message to the person the child was with

Does your child have a cellular phone?

A
Yes

B
No

What did you like most about the study?

What would make the study better?

Powered by Qualtrics
