# Supplementary material for: Automated Adherence Reminders for High Risk Children With Asthma: A Research Protocol
Source: JMIR Res Protoc. 2017 Mar 27;6(3):e48. doi: 10.2196/resprot.6674 (PMC5387114; doi:10.2196/resprot.6674)
Supplement: Multimedia Appendix 2 [file resprot_v6i3e48_app2.pdf]

<u>**Parental-Child Conflict Assessment**</u>

**How much** does your child resist (e.g., complain, fuss, run away, argue, refuse to do it) when it is time to take his/her daily medication?	A Lot	Quite a Bit	Some of the Time	A Little Bit	Not At All

**How often** does your child resist (e.g., complain, fuss, run away, argue, refuse to do it) when it is time to take his/her daily medication?	All of the Time	Most of the Time	Some of the Time	A Little Bit of the Time	Never
